# Ultrasound-Guided Thrombin Injection for Postcatheterization Pseudoaneurysms and Its Extended Indications

**DOI:** 10.3400/avd.oa.21-00071

**Published:** 2022-03-25

**Authors:** Hirotsugu Ozawa, Takao Ohki, Kenjiro Kaneko, Masamichi Momose, Shigeki Hirayama

**Affiliations:** 1 Vascular Surgery, Shin-yurigaoka General Hospital, Kawasaki, Kanagawa, Japan; 2 Division of Vascular Surgery, Department of Surgery, The Jikei University School of Medicine, Tokyo, Japan

**Keywords:** endovascular treatment, pseudoaneurysm, ultrasound-guided thrombin injection, indication, bleeding

## Abstract

**Objective:** Ultrasound-guided thrombin injection (UGTI) is an option for the treatment of postcatheterization pseudoaneurysms. This method is less invasive and less time-consuming compared with other procedures since it can be performed without general anesthesia, skin incision, or occlusion of the artery. Herein, we report on the efficacy of UGTI for postcatheterization bleeding complications.

**Methods:** Postcatheterization bleeding complications include postcatheterization pseudoaneurysm and failed hemostasis. In this study, failed hemostasis was defined as cases in which hemostasis could not be accomplished by 30 min of manual compression following sheath removal. A retrospective study of eight cases in which we performed UGTI for postcatheterization bleeding complications between July 2016 and June 2019 at our institution was performed to evaluate technical success and recurrence of pseudoaneurysm or rebleeding events.

**Results:** Among these eight cases, there were three cases of pseudoaneurysm and five cases of failed hemostasis. In all cases, technical success was achieved without any complications such as distal embolism or allergic reaction. There were no recurrences of pseudoaneurysm or rebleeding events during an average follow-up of 5.25 months.

**Conclusion:** We believe that UGTI is effective not only for postcatheterization pseudoaneurysms but also for failed hemostasis.

## Introduction

Access site complications following endovascular treatment (EVT) include bleeding/hematoma, pseudoaneurysm (PSA), and arteriovenous fistula, in decreasing order of frequency.^1)^ Although rare, some of these are life- and limb-threatening, such as retroperitoneal hemorrhage or arterial occlusion. Thus, it is imperative to secure adequate hemostasis for a good outcome after EVT.

Postcatheterization bleeding complications can lead to tenderness and swelling at the access site, and large hematomas or PSA can cause local compression, leading to neuropathy, deep venous thrombosis, or skin necrosis. In addition, rupture of the PSA can occur when the diameter of the PSA is larger than 3 cm.^2)^ Treatment strategies for postcatherization PSA include ultrasound-guided compression (UGC), ultrasound-guided thrombin injection (UGTI), and open surgical repair.

UGC is a noninvasive option, but this method requires 30 min on average to accomplish hemostasis and may lead to discomfort to the patients.^1)^ As for open repair of postcatheterization PSAs, a skin incision is placed at the affected site, and the arteriotomy can be closed with suture after securing control of arterial flow using a surgical clamp or balloon occlusion. Thus, this is a definitive treatment since hemostasis is achieved under direct vision. However, general anesthesia is usually required, and a high rate of incisional dehiscence and infection has been reported since the incision must be made at an already swollen site.^3)^ Because of its speed and simplicity, UGTI should be considered before proceeding to open repair. However, UGTI is not yet covered by the national health insurance in Japan. Therefore, open repair plays a central role in the treatment of postcatheterization PSA when compression procedures fail.

UGTI has been developed as a percutaneous procedure for postcatheterization PSA.^4–6)^ In this method, thrombin is injected directly into the PSA under ultrasound guidance, enabling instantaneous and complete thrombosis inside the PSA. Thrombosis immediately occurs when a high concentration of thrombin mixes with stagnant blood inside the PSA, leading to conversion of fibrinogen to fibrin. This reaction occurs even in the presence of anticoagulation and/or antiplatelet therapy. UGTI is simple and quick, can alleviate discomfort and pain for patients treated with compression procedures, and also eliminates the risk of wound-related complications since there incision is not needed.

After having introduced UGTI for postcatheterization PSAs in July 2016, we expanded the application of this procedure to cases in which complete hemostasis cannot be achieved within 30 min of compression at the puncture site after removal of the sheath. Hereafter, we refer to such cases of failed hemostasis as failed hemostasis (FH).

## Methods

### Study design

This was a retrospective study performed to investigate the clinical results of UGTI for the treatment of postcatheterization bleeding complications consisting of PSA and FH. All consecutive patients undergoing UGTI for the treatment of postcatheterization PSA and FH at our institution were enrolled in the study. This study was approved by the institutional review board (IRB). Also, UGTI was approved by the IRB since this procedure remains an off-label use of thrombin, which is produced as a topical hemostatic agent and not yet authorized for injection.

### Diagnostic imaging

Diagnostic duplex ultrasound was performed with Xario 100 (Cannon Medical Systems Corporation, Otawara, Tochigi, Japan). Duplex ultrasound imaging was indicated for post-EVT patients who develop tenderness, swelling, or a mass at the access site. A linear transducer was used initially for evaluation, and a convex transducer was reserved for obese patients or those with a large groin PSA or hematoma. Duplex imaging included B-mode imaging for measuring hypoechoic masses, and color flow imaging was used for differentiating a simple hematoma from a PSA as well as for acquiring anatomical information of the arterial defect and the aneurysmal sac. Normally, at our institution, computed tomography angiography (CTA) is added for patients diagnosed with postcatheterization PSA, in order to evaluate extension of a hematoma into the retroperitoneum and rule out the existence of arteriovenous fistula. CTA was also used to recognize the anatomical characterization of the PSA visually by its three-dimensional (3D) reconstructed image.

Regarding FH, ultrasound imaging was indicated if FH was clinically suspected by signs such as swelling of the puncture site or bleeding from the puncture site on the skin immediately after the compression was released. As a side note, we usually performed manual compression to secure hemostasis at the puncture site following EVT during this study period. FH was then confirmed when extravasation from the puncture site was identified in color flow imaging.

### Indications

Nonconservative treatment of postcatheterization PSA was indicated when the PSA was larger than 3 cm or larger than 1 cm with any symptoms of aneurysmal growth. Two vascular surgeons decided whether the PSA should be treated with UGTI or an open repair on a case-by-case basis. For patients with postcatheterization FH, UGTI was indicated when control of bleeding from the puncture site on the skin could be achieved by compression using the ultrasound probe.

Contraindications of UGTI included the following: (1) patients with known allergy to bovine-derived products, (2) PSA with rupture, skin necrosis, infection or arteriovenous fistula, (3) PSA that had already thrombosed, (4) PSA in which the size of the arterial defect was thought to be larger than the diameter of the affected artery, and (5) FH in which bleeding could not be controlled at all by compression with the ultrasound probe.

### Subjects

Between July 2016 and June 2019, 13 cases of postcatheterization bleeding complications after EVT were found at our institution. Among these, eight cases were treated using UGTI and were enrolled in this study.

### Endpoint definition

Technical success was defined as complete obliteration of the cavity of the PSA or the extravascular space in FH.

### UGTI procedure

After informed consent was obtained, the affected site was prepared in a sterile fashion, and local anesthesia was applied with 1% lidocaine infiltrated into the skin. The UGTI procedure was then performed as follows: a three-way stopcock is used, and a bovine thrombin solution (1000 U/ml; Mochida Pharmaceutical, Tokyo, Japan) and saline are drawn up in separate 1-ml syringes and loaded to the stopcock, which is turned off to the thrombin syringe and on to the saline syringe. The stopcock is attached to a 21-gauge needle. Under ultrasound guidance, the tip of the needle is placed into the sac of the PSA. During this time, the tip of the needle should always be visualized in order to avoid inadvertent puncture to the affected artery or accompanying vein. Once blood flows back into the saline syringe, saline is then injected into the sac to confirm placement into the PSA. The stopcock is then turned off to saline and on to the thrombin, and 0.2 ml (200 U) of thrombin is injected, regardless of diameter of the PSA, while observing the blood flow into the sac with color flow imaging. If persistent flow is observed within the sac after a few seconds of observation, an additional 0.1–0.2 ml of thrombin is injected as needed. Completion ultrasound is performed to confirm the obliteration of flow within the PSA and patent native artery. Moreover, the peripheral blood flow is confirmed on pulsation or Doppler flowmeter.

In cases of FH, we first confirmed control of bleeding from the puncture site on the skin by compression with the ultrasound probe. Control of bleeding is necessary before UGTI can be performed. A small extravascular space with arterial flow, which is created by the extravasation in the soft tissue, can be detected in color flow imaging. At this time, appropriate pressure of the probe on the puncture site should be applied to prevent the formation of hematoma or to prevent the growth of hematoma that has already formed. Under ultrasound guidance with appropriate pressure, the tip of the needle is placed into the space, followed by confirming that the blood flows back. Thrombin injection is then performed in the manner described above. Completion ultrasound is performed to confirm thrombosis of the extravascular space and a patent native artery.

After the procedure, 2–4 h of bedrest is ordered with no need of post-UGTI compression. Follow-up duplex ultrasound is obtained twice: 1 day and 1 week after UGTI.

## Results

**Table 1** summarizes the characteristics of the patients, lesions, and procedures.

**Table 1:** Table 1 Characteristics of the patients, lesions, and the procedures

Patient No.	Age	Gender	Primary disease EVT done for	Location	Condition	Diameter of PSA (mm)	Procedure time (min)	Thrombin dose (ml, 1,000 U/ml)
1	70	M	LEAD	CFA	PSA	59	60	4
2	52	M	VA failure	BA	PSA	20	22	0.6
3	74	F	LEAD	CFA	FH	N/A	8	0.8
4	67	M	Cerebral embolism	CFA	PSA	45	15	0.6
5	71	M	LEAD	CFA	FH	N/A	20	5
6	81	M	LEAD	CFA	FH	N/A	10	0.4
7	84	M	LEAD	CFA	FH	N/A	10	1.2
8	86	M	LEAD	SFA	FH	N/A	10	0.8
Mean/median*	73.1±10.3					41.3±16.1	12.5 (8–60)	0.8 (0.4–5.0)

* Mean±standard deviation; median (range) EVT: endovascular treatment; LEAD: lower extremity arterial disease; VA: vascular access; CFA: common femoral artery; SFA: superior femoral artery; BA: brachial artery; PSA: pseudoaneurysm; FH: failed hemostasis; N/A: not applicable

We performed UGTI in eight cases following EVT. Among these eight cases, three PSA and five FH were included. The mean age was 73.1 years (range 52–86 years), and causative EVT were as follows: six cases of EVT for the treatment of lower extremity arterial disease, one case of percutaneous cerebral thrombectomy for cerebral embolism, and one case of vascular access intervention therapy for vascular access failure. Six lesions were located in the common femoral artery (CFA), one in the superficial femoral artery (SFA), and one in the brachial artery (BA). Mean duration from prior EVT to UGTI was 3.6 days (range 0–17 days).

The three PSA had a mean diameter of 41.3±16.1 mm. In cases of FH, the diameter was not applicable since there is no aneurysm sac. The median procedure time was 12.5 min (range 8–60 min). The median thrombin dose was 0.8 ml (range 0.4–5.0 ml), which is equivalent to 800 IU (range 400–5,000 IU). In our first case, 60 min of procedure time was needed because we added angiography during UGTI due to safety issues.

In all eight cases, technical success was achieved without any complications, such as distal embolism or allergic reaction. During an average follow-up of 5.25 months (range 0–30 months), no recurrences of pseudoaneurysm or rebleeding events were observed, and no secondary interventions after UGTI were required.

## Case Presentation

### Case 1 (Table 1, Patient No. 4)

A 67-year-old man developed right-sided hemiplegia and aphasia. Magnetic resonance imaging demonstrated cerebral infarction in the area of the left middle cerebral artery. After being diagnosed with acute cerebral embolism due to atrial fibrillation, he underwent percutaneous thrombectomy with a right femoral approach. Although the thrombectomy was successful, his right-sided hemiplegia and aphasia persisted postoperatively. Moreover, he was taking edoxaban postoperatively.

**Figure 1:**
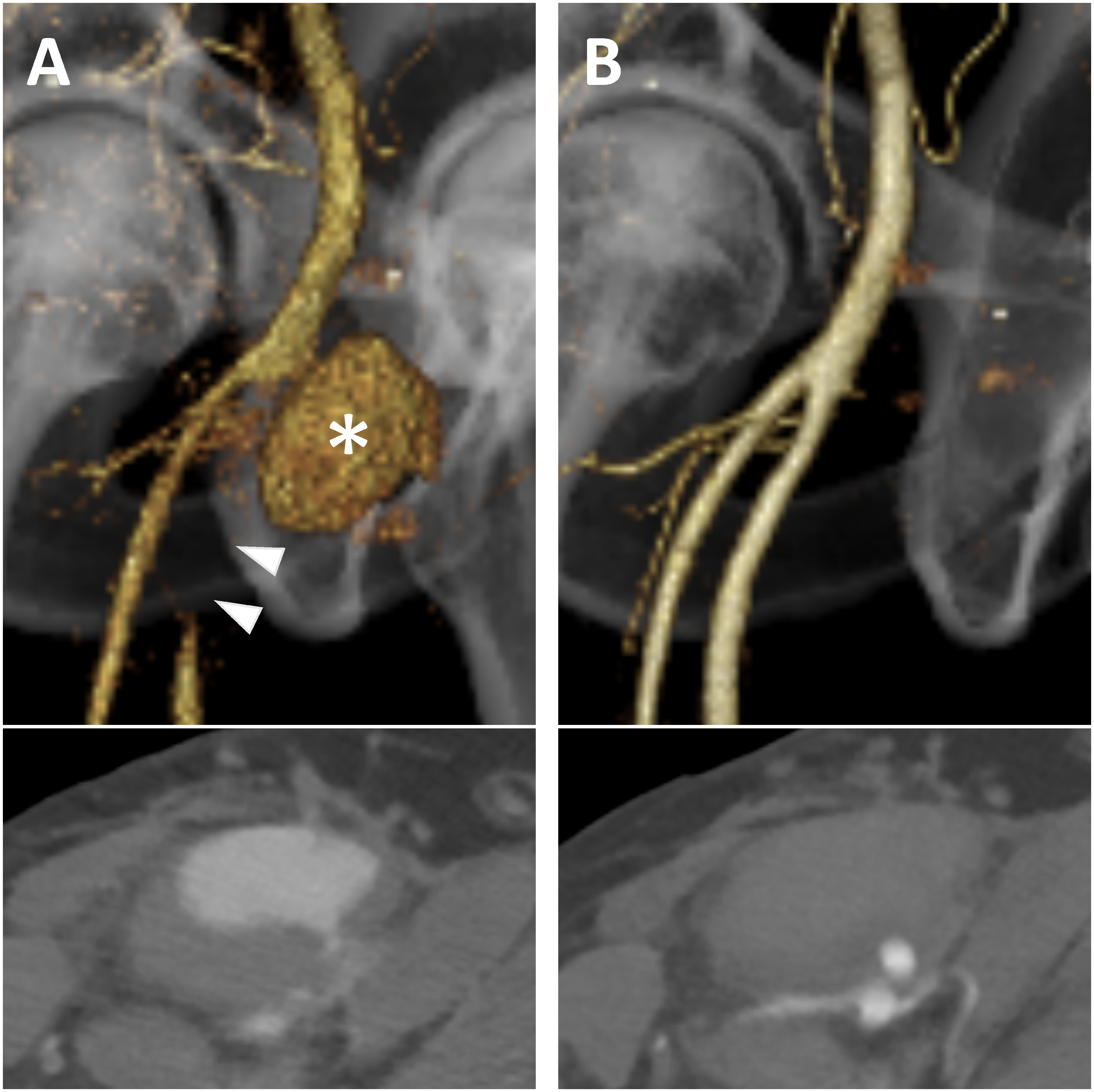
Fig. 1 (**A**) Digitally recreated three-dimensional (3D) image of computed tomography angiography shows the pseudoaneurysm of the common femoral artery (asterisk), complicated with severe stenosis of the superficial femoral artery due to compression by the pseudoaneurysm (arrowheads). (**B**) This is a 3D image 5 days after the ultrasound-guided thrombin injection, showing disappearance of the pseudoaneurysm and resolution of the external compression to the superficial femoral artery.

On postoperative day 17, a pulsatile mass in the right groin was found, and ultrasound sonography and CTA revealed a PSA of 45 mm in diameter in the right CFA. CTA also demonstrated severe stenosis of the proximal part of the right SFA due to external compression caused by this large PSA (**Fig. 1A**). We performed UGTI for the PSA using 0.6 ml (600 IU) of thrombin solution, with 15 min of procedure time. CTA performed 5 days after UGTI showed a completely thrombosed PSA without any contrast inside the sac of the PSA, and also stenosis of the right SFA, which had been caused by the PSA, had disappeared (**Fig. 1B**). One month after UGTI, he transferred to a rehabilitation hospital for further rehabilitation. No recurrence of the PSA was detected during this 1-month period.

### Case 2 (Table 1, Patient No. 6)

An 81-year-old male admitted to our hospital with lifestyle-limiting claudication of the right leg (Rutherford class 2). He was a current smoker and had a history of hypertension and peripheral artery disease with previous EVT on his left leg 9 months prior to admission. He was taking aspirin. In this admission, he underwent EVT with stenting in the right external iliac artery and drug-coated balloon angioplasty in the right SFA via a 6 F guiding sheath placed into the left CFA.

**Figure 2:**
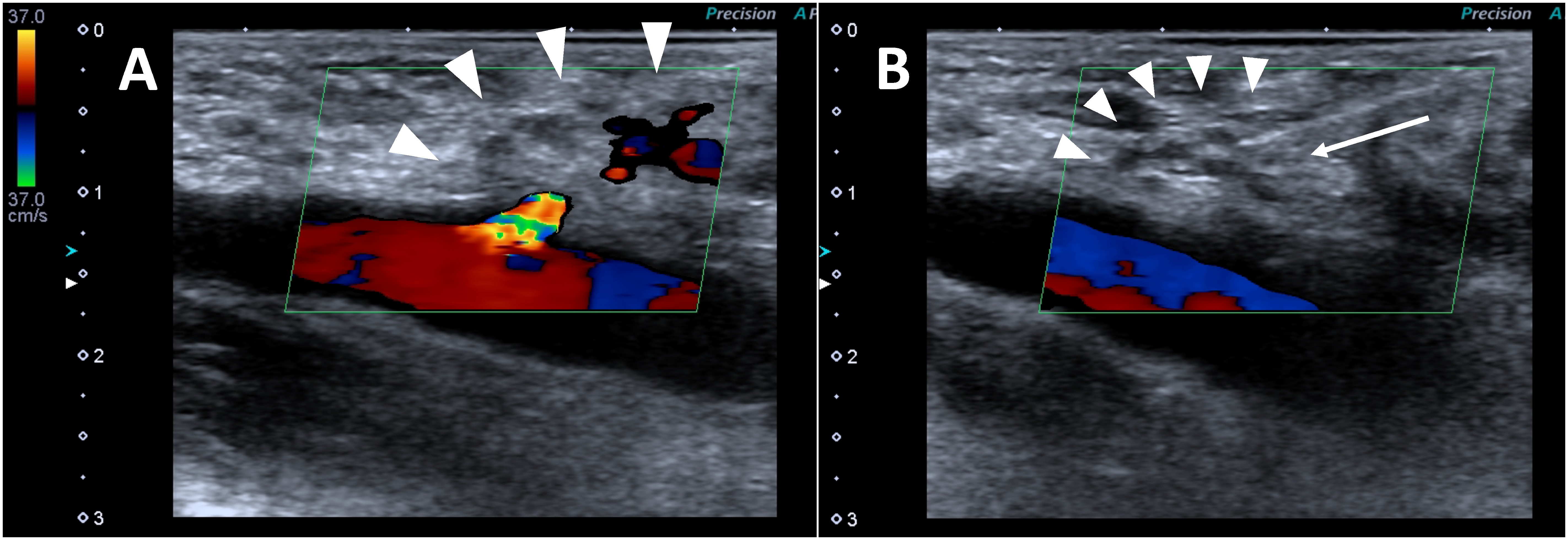
Fig. 2 (**A**) A small extravascular space with arterial flow via the arterial defect was seen in the color flow imaging (arrowheads). (**B**) A 21 G needle was placed into the extravascular space (arrow). Once blood flowed back, thrombin injection was performed, resulting in immediate thrombosis of the extravascular space (arrowheads).

After removing the sheath, manual compression was applied at the puncture site. However, 30 min of manual compression failed to achieve hemostasis since bleeding from the puncture site on the skin was observed if we reduced the compression. Therefore, the decision was made to proceed with UGTI, and manual compression was converted to compression using the ultrasound probe. A small extravascular space with arterial flow via the arterial defect was seen in color flow imaging (**Fig. 2A**). A 21-gauge needle was then placed into this space under ultrasound guidance. Once blood flowed back, a total of 0.4 ml (400 IU) of thrombin solution was injected, resulting in immediate thrombosis of the extravascular space (**Fig. 2B**). The postoperative course was uneventful with no recurrence of rebleeding events, and he was discharged 1 day after the procedure.

## Discussion

We performed UGTI for postcatheterization bleeding complications in eight patients with a technical success rate of 100% and no complications or new bleeding events. UGTI takes only about 15 min to perform. We believe that UGTI is effective and feasible not only for postcatheterization PSAs but also FH after sheath removal.

In cases of FH, there is a disruption in the arterial wall similar to PSA, but there is no aneurysmal sac at all. Until November 2018, we offered UGC to such FH patients. However, it is lengthy, painful, and uncomfortable. Moreover, some of the patients cannot endure this procedure to the point of completion, necessitating open surgical repair. Therefore, we introduced UGTI for FH. Using this procedure, we can achieve instantaneous thrombosis of the extravascular space. In fact, five cases of FH in our series showed excellent results with technical success of 100% and the mean procedure time of 13.6 min, without any procedure-related complications or post-UGTI rebleeding events.

When we encounter a case of FH, a closure device cannot be used since the guide wire has already been removed by this stage. Therefore, UGC and open surgical repair are usually the only options. Of course, it is possible that hemostasis may be achieved with an additional 30 min of UGC. However, we believe that UGTI is preferable to UGC due to its higher technical success and shorter procedure time, with less discomfort to the patient. In cases where the extravascular space disappears due to compression with the ultrasound probe, we slightly reduce the compression to visualize the extravascular space and facilitate needle placement, while keeping sufficient compression so that the hematoma does not grow.

Percutaneous thrombin injection for the treatment of postcatheterization PSA was first reported in 1986.^4)^ This method was originally performed under angiographic guidance and was later modified to utilize ultrasound guidance as we do today,^5,6)^ and it has become increasingly popular. According to a study of 1,329 cases of UGTI, the primary success rate is very high at 91%–100% (97.5% on average).^2)^ Although there is a recurrence rate of 0%–9% (3.3% on average), recurrent PSAs can be also treated with a second injection with excellent results.^2)^ The overall complication rate from UGTI is 1.3%, including distal embolization, arterial thrombosis, and allergic reactions. Embolization and arterial thrombosis are mostly due to inadvertent puncture of the artery or vein or injection into the neck of the PSA.^2)^ In this procedure, distal embolization is unlikely to happen. This is because thrombosis only occurs when a high concentration of thrombin mixes with relatively stagnant blood and thrombin at the PSA site, and if thrombin were somehow to leak into the artery, it would be effectively diluted in the circulating blood and neutralized by the anticoagulant factors such as antithrombin III and thrombomodulin.^7)^ However, this is not the case when a large volume of thrombin is administered rapidly into the native artery. Therefore, precise placement and constant visualization of the tip of the needle, slow and incremental injection of thrombin, and avoiding excessive dosage amounts are indispensable. Severe allergic reaction after thrombin injection is extremely rare.^8)^

In our experiences and those of others, a dose of approximately 1,000 units of thrombin is required to induce thrombosis.^9)^ In the first case in our series, the patient was severely obese and had a huge PSA, rendering it difficult to visualize the PSA with ultrasound imaging and resulting in the use of 4,000 units of thrombin. The other two PSAs could be treated with an appropriate dose of thrombin. As for the cases of FH, most cases just required approximately 1,000 units. However, 5,000 units were needed in one case, in which compression with the probe was not effective enough to control bleeding completely. This was due to obesity and a relatively high puncture site, rendering the extravascular space larger, more complicated, and multilocular. As a result, injection into several chambers was required, eventually using as much as 5,000 units. In general, we believe that FH can be treated with UGTI in a similar fashion to PSA. On the other hand, in cases where compression with the probe is untenable, a large dose of thrombin is likely to be required; thus extra care should be taken. In addition, physicians should be flexible in their approach and consider open surgery where UGTI is not suitable.

While performing UGTI for traumatic PSA has been reported, the authors argued that traumatic indications should be selective since traumatic arterial defects can be large.^10)^ In addition, there is a report of thrombosis of an artery after successful UGTI of an iatrogenic brachial PSA in an infant patient.^11)^ However, it is likely that this was due to the very small size of the artery and the relatively large size of the defect, as has been reported on.^10)^ We believe that small defect size relative to the size of the affected artery is the primary predictive factor for successful UGTI, and keeping this in mind is essential to achieving successful treatment and avoiding recurrence and complications.

Although inflating a balloon at the site of the neck of a PSA in order to prevent leakage of thrombin into the artery has been described,^12)^ an additional puncture is necessary to do this, and we generally avoid this to limit the risk of further bleeding complications. Moreover, unlike the standard method (described herein) in which injection is finished as soon as persistent flow inside the PSA disappears, using a balloon creates the risk of administering excess dose of thrombin. Thus, we do not usually utilize balloon protection in our practice. However, balloon protection might be beneficial in cases of traumatic or carotid PSAs, where it is paramount to avoid the potential risk of distal embolization or arterial thrombosis.^13,14)^

During the same period as this study, we performed open surgical repair for postcatheterization PSA in five cases, but we had no cases in which we performed surgery for FH. Of these, all the cases were PSAs that occurred after percutaneous coronary interventions, and two were located in the SFA, two in the radial artery, and one in the BA. All these cases were encountered during the early phase of this study. In retrospect, the two SFA cases could probably have been treated by UGTI. In the BA case, the PSA had already been thrombosed before the patient was referred to us, but the neurological symptoms in the affected limb had not improved, so we decided to undertake surgical repair. Regarding radial PSAs, we favor surgical repair because it can be usually performed under local anesthesia, and it is relatively easy to expose the artery.^15)^ While an acceptable outcome of UGTI for a radial PSA has been described,^16)^ we believe that caution should be taken when considering UGTI for PSAs in the radial artery (RA) because the RA has a relatively smaller diameter.

There is a learning curve in performing UGTI, so when expanding its indications, except perhaps for postcatheterization femoral PSAs, the practitioner should at least be proficient at performing UGTI for femoral PSAs, and special care must be taken regarding patient selection. To the best of our knowledge, this is the only article that describes the use of UGTI for FH immediately after EVT. We believe that UGTI of FH is also safe and effective, and UGTI can be a new option for the treatment of FH. Since our sample size was very small, and a selection bias existed in this study because we had no standard protocol for choosing optimal candidates for UGTI, accumulation of further cases is necessary to establish the effectiveness of UGTI for the treatment of FH.

## Conclusion

It is obvious that UGTI for the treatment of postcatheterization PSAs is safe and effective. In addition, we have demonstrated that UGTI might also be a safe and effective treatment for FH, which is supported by the excellent results in this study.
